# Stigmatization in Patients With Psoriasis: A Mini Review

**DOI:** 10.3389/fimmu.2021.715839

**Published:** 2021-11-15

**Authors:** Hanlin Zhang, Zihan Yang, Keyun Tang, Qiuning Sun, Hongzhong Jin

**Affiliations:** Department of Dermatology, State Key Laboratory of Complex Severe and Rare Diseases, National Clinical Research Center for Skin and Immune Diseases, Peking Union Medical College Hospital, Chinese Academy of Medical Sciences and Peking Union Medical College, Beijing, China

**Keywords:** stigmatization, psoriasis, questionnaires, biologics, immunology

## Abstract

Psoriasis is a chronic and recurrent immune-related skin disease that often causes disfigurement and disability. Due to the visibility of lesions in patients and inadequate understanding of dermatology knowledge in the general public, patients with psoriasis often suffer from stigma in their daily lives, which has adverse effects on their mental health, quality of life, and therapeutic responses. This review summarized the frequently used questionnaires and scales to evaluate stigmatization in patients with psoriasis, and recent advances on this topic. *Feelings of Stigmatization Questionnaire*, *Questionnaire on Experience with Skin Complaints*, and *6-item Stigmatization Scale* have been commonly used. The relationship between sociodemographic characteristics, disease-related variables, psychiatric disorders, quality of life, and stigmatization in patients with psoriasis has been thoroughly investigated with these questionnaires. Managing the stigmatization in patients with psoriasis needs cooperation among policymakers, dermatologists, psychologists, psychiatrists, researchers, and patients. Further studies can concentrate more on these existing topics, as well as other topics, including predictors of perceived stigmatization, stigmatization from non-patient groups, influence of biologics on stigmatization, and methods of coping with stigmatization.

## Introduction

Psoriasis is a chronic and recurrent inflammatory skin disease characterized by clearly demarcated, scaly, erythematous plaques (1–4). Beyond the conventional definition of a skin disorder, psoriasis is now considered a systemic disease (5). Patients with psoriasis may be affected in other organs, such as joint (6) and cardiovascular system (7). Moreover, patients with psoriasis are susceptible to a variety of psychiatric disorders, including depression, anxiety (8), bipolar mood disorder (9), personality disorder (10), and cognitive impairment (11). Psoriasis may place a heavy physical and psychological burden on patients, negatively affecting patients’ health status, private lives, and professional careers (12). Psoriasis could substantially diminish the quality of life in physical, emotional, and social functioning (13, 14).

**Figure 1:**
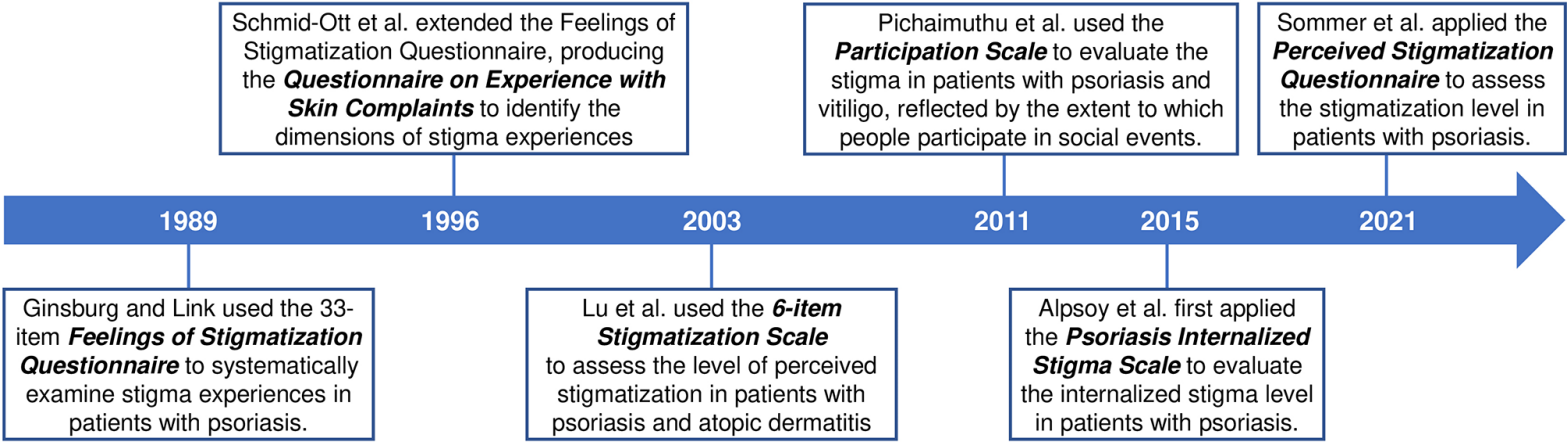
Commonly used questionnaires and scales to evaluate the stigmatization in patients with psoriasis.

Stigmatization was defined as the assignment of biological or social discrediting perceptions to a person, distinguishing a person from others of a society (15, 16). The feeling of stigmatization is common in dermatologic patients, such as psoriasis, vitiligo, and leprosy, mainly because of visible skin lesions, insufficient public understanding of the diseases, and other cultural or social factors (15, 17). Back in the 1950s, Susskind and McGuire reported that psoriasis patients might be exposed to curiosity, hostility, and disgust, because of their “unclean skin” and public concerns about infectivity (18). The publicity of “psoriasis as non-infectious disease” could reduce the burden of these patients (19). In 2018, a global survey included 8,338 patients with moderate-to-severe psoriasis from 31 countries (20). 84% of the respondents experienced psoriasis-related discrimination and/or humiliation, which had negative effects on work, intimacy, and health status. Psoriasis patients may experience social and psychological difficulties in their daily lives, especially when they need to expose their bodies (21). Patients with psychological distress may lose hope and feel out of control over the disease, impairing the response to their therapies (21, 22). In recent years, more and more studies have been conducted on the relationship between stigmatization, sociodemographic characteristics, disease-related variables, and psychiatric disorders in patients with psoriasis. Both dermatology-specific and disease-specific questionnaires can be utilized to evaluate the stigmatization level of psoriasis patients (16, 23). This review aims to summarize these frequently used questionnaires and scales (**Figure 1**), and recent advances on stigmatization in patients with psoriasis, which may facilitate the understanding of this topic and pave the way for further research.

## Feelings of Stigmatization Questionnaire

In 1989, Ginsburg and Link, using “*Feelings of Stigmatization Questionnaire*”, systematically examined stigma experiences in 100 psoriasis patients, and identified six dimensions of stigma (24). This questionnaire is a disease-specific questionnaire consisting of 33 items. The six dimensions were “anticipation of rejection, feeling of being flawed, sensitivity to others’ attitudes, guilt and shame, secretiveness, and positive attitudes”. Stigmatization may result in poor compliance and worsening status. The emphasis on stigmatization in patients with psoriasis has important implications, not only for their quality of life, but also for the clinical management of them.

Several studies used *Feelings of Stigmatization Questionnaire* to evaluate the stigmatization in patients with psoriasis. In 1993, Ginsburg and Link also found that 19% of psoriasis patients experienced episodes of gross rejection due to their skin disease, mainly from a gym, pool, hairdresser, or job (25). Rejection experiences may induce the feeling of being stigmatized and bring about adverse effects on emotions and occupations. Zięciak and colleagues used *Feelings of Stigmatization Questionnaire* and *Beck Depression Inventory*, and found the correlation between the feelings of stigmatization and depressive symptoms in patients with psoriasis (26). In 2017, Hawro and colleagues assessed the stigmatization of 115 psoriasis patients using the *Feelings of Stigmatization Questionnaire*, and found that lesions on the back of hands, rather than overall disease severity, were associated with higher stigmatization levels (12). One possible explanation for this association was the fear of infection, especially when shaking hands, being touched, or touching the same objects. Stigmatization appeared to predict the quality of life impairment best among all analyzed variables. In 2020, Jankowiak and colleagues explored whether sociodemographic variables were related to the stigmatization and quality of life of patients with psoriasis (27). They used the 33-item *Feelings of Stigmatization Questionnaire* and *Dermatology Life Quality Index* (DLQI) to evaluate the level of stigmatization and quality of life, respectively. Authors found that gender and age correlated with different domains of stigmatization, and quality of life correlated significantly with two domains of stigmatization.

## Questionnaire on Experience With Skin Complaints

In 1996, Schmid-Ott and colleagues adopted and extended the *Feelings of Stigmatization Questionnaire*, producing the *Questionnaire on Experience with Skin Complaints (* 28). It was used to identify the dimensions of stigma experience in 187 psoriasis patients. Five factors were identified, including self-esteem, retreat, rejection, composure, and concealment. This questionnaire could assess the stigma experiences of patients with psoriasis and other skin diseases. The same research group also evaluated its validity and concluded that it was valid and reliable in evaluating stigma feelings in atopic dermatitis and psoriasis patients (29). In 2003, the short version of the *Questionnaire on Experience with Skin Complaints* was used for the first time by the research group (30). The short form with 23 items was more economical and had a satisfying Cronbach’s alpha, indicating good validity. Four dimensions of the questionnaire were confirmed by factor analysis, including impairment of self-esteem and withdrawal, rejection experienced, concealment, and composure. In psoriasis and atopic dermatitis patients, the *Questionnaire on Experience with Skin Complaints* was found to have a middle-high correlation with DLQI.

As a dermatology-specific instrument, *Questionnaire on Experience with Skin Complaints* has also been commonly used to evaluate the stigmatization in patients with psoriasis. In 2013, Böhm and colleagues investigated the relations between disease severity, gender, stigmatization, and quality of life (31). The stigmatization levels of 381 patients were measured with the *Questionnaire on Experience with Skin Complaints*. Higher severity of psoriasis was associated with higher stigmatization and lower skin-related quality of life. Men and women experienced different social impacts, but stigmatization affected the quality of life with similar degrees in both genders. In 2014, Bangemann and colleagues adopted the short version of the *Questionnaire on Experience with Skin Complains* and identified stigmatization as a major predictor of quality of life (8). Quality of life was further the strongest predictor of depression and anxiety.

## 6-item Stigmatization Scale

In 2003, a four-point Likert scale was used to assess the level of perceived stigmatization due to skin disease with the following six items: not attractive due to the skin disease, others staring at the skin disease, others feeling uncomfortable touching me due to the skin disease, others regarding the skin disease as contagious, others avoiding me due to the skin disease, others sometimes making annoying comments about the skin disease (32). Cronbach’s alpha for this 6-item stigmatization scale was over 0.8 in patients with psoriasis and atopic dermatitis, which represented good internal consistency. Authors investigated the level of perceived stigmatization and predictors of stigmatization in patients with psoriasis and atopic dermatitis, and found that perceived helplessness was the strongest predictor of the experience of stigmatization in both groups. Two limitations should be emphasized. The use of self-report measures might underestimate the contribution of clinical status to stigmatization. Besides, other psychological factors possibly relevant to chronic skin diseases should also be considered.

This *6-item stigmatization scale* is a dermatology-specific scale, which has been commonly used with *Feelings of Stigmatization Questionnaire* and *Questionnaire on Experience with Skin Complaints*. Studies also focused on disease severity, quality of life, and feeling of stigmatization with these stigmatization questionnaires and other assessments, including *Psoriasis Area and Severity Index* (PASI), *Acceptance of Illness Scale* (AIS), *Satisfaction with Life Scale* (SWLS), and DLQI (33, 34). In a cross-sectional study evaluating stigmatization in Arabic psoriatic patients, researchers found that most patients showed feelings of stigmatization due to psoriasis, and face involvement appeared to be the only independent factor influencing the level of stigmatization (33). Łakuta and colleagues investigated the associations between site of skin lesions and depression, social anxiety, body-related emotions and feelings of stigmatization in patients with psoriasis (35). Several body regions were identified as ‘sensitive’, where psoriasis was associated with negative mental health. Another study included 166 patients with plaque psoriasis to determine their level of stigmatization and the association between the level of stigmatization and other factors (36). Men, countryside dwellers, unmarried persons, patients with a longer history of the disease had significantly higher stigmatization levels than women. In 2021, Kowalewska and colleagues evaluated the effect of disease severity on the quality of life and sense of stigmatization in psoriasis patients (37). Authors concluded that the severity of the disease was the strongest determinant of the quality of life, and the levels of stigmatization correlated significantly with PASI scores.

## Participation Scale

In 2011, a cross-sectional study evaluated the stigma among vitiligo and psoriasis patients in India (38). The key issue of stigma is that it excludes people from participating in social events. The *Participation Scale* used in stigma reduction, rehabilitation, and social integration programs was applied to measure the extent to which people participated in social events (39). Researchers found that psoriasis patients faced more restrictions in their daily lives, 28% of whom participated minimally in domestic and social life, and 2.7% of whom had extreme participation restrictions.

## Psoriasis Internalized Stigma Scale

In 2015, the *Psoriasis Internalized Stigma Scale* was first applied to psoriasis patients (40). Internalized stigma includes endorsing negative feelings and beliefs and applying them to oneself, decreasing self-esteem and life-satisfaction, increasing depression and suicidality, and causing difficulty in coping with the illness (41). The *Psoriasis Internalized Stigma Scale* was proved to be reliable and valid with Cronbach’s alpha 0.89. The *Psoriasis Internalized Stigma Scale* was significantly correlated with the *Dermatology Quality of Life Index* (DQoL) scores (r=0.726). A multi-centered, cross-sectional study included 1,485 patients, investigated the internalized stigma state of patients with psoriasis, and identified the factors influencing internalized stigma using *Psoriasis Internalized Stigma Scale (* 41). Authors found that disease severity, involvement of visible body parts, genital area, folds or joints, poorer quality of life, negative perceptions of general health and psychological illnesses were associated with high levels of internalized stigma. Another comparative multi-centered study investigated the internalized stigma in pediatric psoriasis patients (42). Internalized stigma in pediatric patients was associated with poorer quality of life, general health, and psychological illnesses. Unlike adults, internalized stigma in pediatric patients was mainly determined by psoriasis itself, rather than disease severity or involvement of visible body parts, genital area, or folds.

## Perceived Stigmatization Questionnaire

In 2021, Sommer and colleagues used the *Perceived Stigmatization Questionnaire* to assess the level of stigmatization in patients with psoriasis, and found that stigmatization experiences were positively associated with younger age, disease severity, scratching behaviors, dysmorphic concerns, and treatment benefits (43). The *Perceived Stigmatization Questionnaire* is a 5-point Likert scale (never, almost never, sometimes, often, always) with 21 items in six categories, the reliability of which was previously confirmed with Cronbach’s alpha 0.93 in a group of burn survivors (44).

## Discussion

Psoriasis is a chronic and recurrent immune-mediated skin disease that often causes disfigurement and disability in patients (1, 45–48). Due to the inadequate popularization of dermatology knowledge, patients with psoriasis often receive stigmatization in their work and life, negatively affecting their quality of life and even leading to mental illnesses such as anxiety and depression. Over the past few decades, a variety of questionnaires have been developed or applied to assess the stigmatization of patients with psoriasis (16). *Feelings of Stigmatization Questionnaire*, *Questionnaire on Experience with Skin Complaints*, and *6-item Stigmatization Scale* are commonly used in stigmatization assessment among psoriasis patients recently, based on which many essential conclusions on stigmatization have been drawn.

It was proved that several factors could predict the stigmatization level of patients with psoriasis, including sociodemographic variables, disease-related variables, and personality variables. Among the sociodemographic variables, gender correlated with stigmatization level in some studies (27, 49), while in others not (26, 34). Lower education (50), lack of professional knowledge (51), and countryside residence (52) were associated with higher levels of stigmatization, which might be secondary to inadequate understanding of psoriasis. The popularization of psoriasis characteristics among the public, particularly its non-infectious nature, might improve the stigmatized condition and the acceptance of patients (53). Among the disease-related variables, age of onset was proved to correlate with feelings of stigmatization, and patients with early-onset age were more susceptible (24, 54, 55). Psoriasis-related social stigma affected individuals in earlier adulthood more negatively, who just established their social relationships and contacted a wider range of people (55). Thus, extra attention should be paid to children and adolescent patients by physicians. Besides, skin lesion distribution and disease severity appeared to be associated with stigmatization in some studies (12, 31, 35, 50). Skin lesions at the exposed areas were associated with higher stigmatization levels, increasing the risk of social exclusion and hurting patients’ quality of life. Therefore, the lesion distribution should be considered together with overall disease severity during the treatment, and psoriasis at the exposed areas required special attention. Moreover, type D personality was found correlated with stigmatization, probably because of the inhibited emotion or behavior caused by fear of disapproval (50). As a result, type D personality screening might be needed to assess the stigmatization level among the patients. Investigation on the proper predictors provided a framework for patients with a high risk of stigmatization, promoting screening and intervention procedures for further implementation of tailored evidence-based treatment (50).

Nowadays, several studies on psoriasis stigmatization have also been conducted based on non-patient groups. In 2018, Sommer and colleagues investigated the public awareness of psoriasis in German (56). 9% of the surveyed thought psoriasis was communicable, and 27% were unwilling to have a personal relationship with the affected. The same group also assessed prejudice and stigmatization of psoriasis patients in the general German population (57). People with psoriasis were considered disadvantaged and disgusting by a majority of the surveyed. Most participants did not want to touch psoriasis patients, and some thought patients should ‘take better care of themselves’. Age, gender, and education level were associated with some prejudiced attitudes. In 2019, Pearl and colleagues compared the stigmatizing attitudes towards psoriasis among laypersons and medical students (51). The medical trainees reported fewer stigmatizing attitudes than laypersons, which indicated that an educational campaign about psoriasis among the public might help reduce the stigmatization of psoriasis patients.

Topical agents are the primary option for patients with mild psoriasis, while systemic treatments are the mainstay for moderate to severe psoriasis. In 2005, Nijsten and colleagues assessed psoriasis patients’ satisfaction with four traditional systemic treatments (58). Less than 40% of the users were very satisfied with any of the four therapies. With a better understanding of pathogenesis, dermatologists began to create new pathogenesis-based therapies, especially various biologics. Over the past two decades, a variety of biologics have been proved to be safe and effective in the treatment of psoriasis, especially in moderate-to-severe psoriasis (13, 59, 60). Biologics are recommended by the American Academy of Dermatology-National Psoriasis Foundation guidelines as a first-line treatment option for moderate-to-severe plaque psoriasis. The introduction of biologics increased medication adherence and treatment satisfaction (61), and improved patients’ quality of life (62). In 2013, Tennvall and colleagues reported that patients receiving biological treatment of 12 months showed the highest satisfaction and the lowest DLQI score, compared to the topical treatment group and systemic and/or biological <12 months treatment group (62). In 2015, Schaarschmidt and colleagues compared the patients’ satisfaction with four treatment modalities (63). Participants obtaining biologics were the most satisfied, with ustekinumab receiving the highest *Treatment Satisfaction Questionnaire for Medication* score. In 2018, Ichiyama and colleagues concluded that treatment satisfaction was significantly correlated with disease severity and quality of life impairment (61). Patients who received biologics were more satisfied than those who received non-biologics, indicating better skin condition and quality of life. Because of the correlation between disease severity, stigmatization, and quality of life (31), the application of biologics probably decreases the level of perceived stigmatization in patients with psoriasis. More research is needed in the future, focusing on the effects of biologics on the stigmatization level in patients with psoriasis.

Methods of helping patients cope with the stigmatization of psoriasis should be emphasized, and necessary psychological and social support should be provided. The potential influence of stress on disease onset and severity was shown in a subgroup of psoriasis patients (64). Therefore, psoriasis management should address both physical and psychosocial aspects. Other forms of support have been emphasized in the treatment of psoriasis, such as relaxation techniques, cognitive-behavioral therapy, and support groups (65). The cooperation of dermatologists and psychiatrists is highly warranted to manage the stigmatization in psoriasis patients. Germen has translated the WHA resolution to a “Destigmatization” program for visible chronic skin diseases (66). Such activities are of great significance in regions where psoriasis is highly prevalent and stigmatized, and require cooperation among policymakers, dermatologists, psychologists, psychiatrists, researchers, and patients (67).

This review summarized the frequently used questionnaires and scales to evaluate stigmatization in patients with psoriasis, and recent advances on this topic. *Feelings of Stigmatization Questionnaire*, *Questionnaire on Experience with Skin Complaints*, and *6-item Stigmatization Scale* have been commonly used. The relationship between sociodemographic characteristics, disease-related variables, psychiatric disorders, quality of life, and stigmatization in patients with psoriasis has been thoroughly investigated with these questionnaires. Two limitations should be emphasized. First, the strengths and weaknesses of some questionnaires, and the rationale for developing additional questionnaires were not summarized, because the original articles using these questionnaires focused little on these topics, and few articles compared different questionnaires in evaluating stigmatization in patients with psoriasis. Second, selection bias should also be considered, not only on the selected patients in these studies, but also on the selected articles in this review. Further studies can concentrate more on these existing topics, as well as other topics, including predictors of perceived stigmatization, stigmatization from non-patient groups, influence of biologics on stigmatization, and methods of coping with stigmatization.

## Author Contributions

HZ, ZY, and KT contributed equally to this manuscript. HZ designed the study and wrote the manuscript. ZY wrote the manuscript. KT and QS made the figure and revised the manuscript. HJ supervised the study. All authors contributed to the article and approved the submitted version.

## Funding

This paper was supported by the National Natural Science Foundation of China (81773331 and 82073450), and the National Key Research and Development Program of China (2016YFC0901500).

## Conflict of Interest

The authors declare that the research was conducted in the absence of any commercial or financial relationships that could be construed as a potential conflict of interest.

## Publisher’s Note

All claims expressed in this article are solely those of the authors and do not necessarily represent those of their affiliated organizations, or those of the publisher, the editors and the reviewers. Any product that may be evaluated in this article, or claim that may be made by its manufacturer, is not guaranteed or endorsed by the publisher.
